# Closure or medical therapy of patent foramen ovale in cryptogenic stroke: prospective case series

**DOI:** 10.1186/s42466-021-00114-3

**Published:** 2021-04-01

**Authors:** Sven Poli, Elisabeth Siebert, Joshua Mbroh, Khouloud Poli, Markus Krumbholz, Annerose Mengel, Simon Greulich, Florian Härtig, Karin A. L. Müller, Wolfgang Bocksch, Meinrad Gawaz, Ulf Ziemann, Christine S. Zuern

**Affiliations:** 1 Department of Neurology & Stroke, Eberhard-Karls University, Hoppe-Seyler-Str. 3, 72076 Tübingen, Germany; 2 Hertie Institute for Clinical Brain Research, Eberhard-Karls University, Tuebingen, Germany; 3 Department of Cardiology, Eberhard-Karls University, Tuebingen, Germany; 4 Cardiology Division, Department of Medicine, University Hospital, and Cardiovascular Research Institute, Basel, Switzerland

**Keywords:** Secondary stroke prevention, Patent foramen ovale, PFO-closure, Cryptogenic stroke, Embolic stroke of undetermined source, ESUS

## Abstract

**Background:**

Results of randomized controlled trials (RCT) do not provide definite guidance for secondary prevention after ischemic stroke (IS)/transient ischemic attack (TIA) attributed to patent foramen ovale (PFO). No recommendations can be made for patients > 60 years. We aimed to compare interventional and medical PFO-management in cryptogenic IS/TIA patients, including patients > 60 years.

**Methods:**

Prospective case series including consecutive cryptogenic IS/TIA patients with PFO at Tuebingen university stroke unit, Germany. ‘PFO-closure’ was recommended in patients ≤70 years when featuring high-risk PFO (i.e., with atrial septal aneurysm, spontaneous, or high-grade right-to-left shunt during Valsalva). Primary (recurrent IS/intracranial hemorrhage) and secondary endpoints (e.g., disability) were assessed during ≥1-year follow-up; planned subgroup analyses of patients ≤60/> 60 years.

**Results:**

Among 236 patients with median age of 58 (range 18–88) years, 38.6% were females and median presenting National Institutes of Health Stroke Scale score was 1 (IQR 0–4). Mean follow-up was 2.8 ± 1.3 years. No intracranial hemorrhage was observed. Recurrent IS rate after ‘PFO-closure’ was 2.9% (95%CI 0–6.8%) and 7% (4–16.4) in high-risk PFO patients ≤60 (*n* = 103) and > 60 years (*n* = 43), respectively, versus 4% (0–11.5) during ‘medical therapy alone’ MTA (*n* = 28). 42 low-risk PFO patients treated with MTA experienced no recurrent IS/TIA.

**Conclusions:**

In our real-world study, IS recurrence rate in ‘PFO-closure’ high-risk PFO patients ≤60 years was comparable to that observed in recent RCT. High-risk PFO patients > 60 years who underwent PFO-closure had similar IS recurrence rates than those who received MTA. MTA seems the appropriate treatment for low-risk PFO.

**Trial registration:**

ClinicalTrials.gov, registration number: NCT04352790, registered on: April 20, 2020 – retrospectively registered.

**Supplementary Information:**

The online version contains supplementary material available at 10.1186/s42466-021-00114-3.

## Introduction

Approximately 25% of humans have a patent foramen ovale (PFO). In otherwise healthy individuals, PFO is not associated with increased risk of ischemic stroke (IS)/transient ischemic attack (TIA) [7, 21]. In patients with cryptogenic IS, however, PFO prevalence is almost 60% [10, 14–16]. Studies have therefore suggested a causative link between PFO and IS/TIA, especially in young patients, patients with atrial septal aneurysm (ASA) or substantial shunt size [5, 18]. The initial randomized controlled trials (RCT; CLOSURE I [9], RESPECT [6], and PC [20]) investigating the role of transcatheter PFO-closure for secondary stroke prevention, however, were unable to demonstrate benefit of ‘PFO-closure’ compared to ‘medical therapy alone’ (MTA). In contrast, results of the more recent RCT (CLOSE [19], REDUCE [29], and DEFENSE-PFO [17]) and the extended follow-up of RESPECT [26] showed that – compared to MTA – PFO-closure can prevent recurrent IS/TIA in patients ≤60 years. Importantly, CLOSE [19] and DEFENSE-PFO [17] solely included patients with ASA or large shunt size, and latter subgroups were the driving force behind positive results in REDUCE [29] and RESPECT [26]. Furthermore, benefit of PFO-closure is only given with double-disk but not with umbrella-clamshell devices [27].

**Fig. 1:**
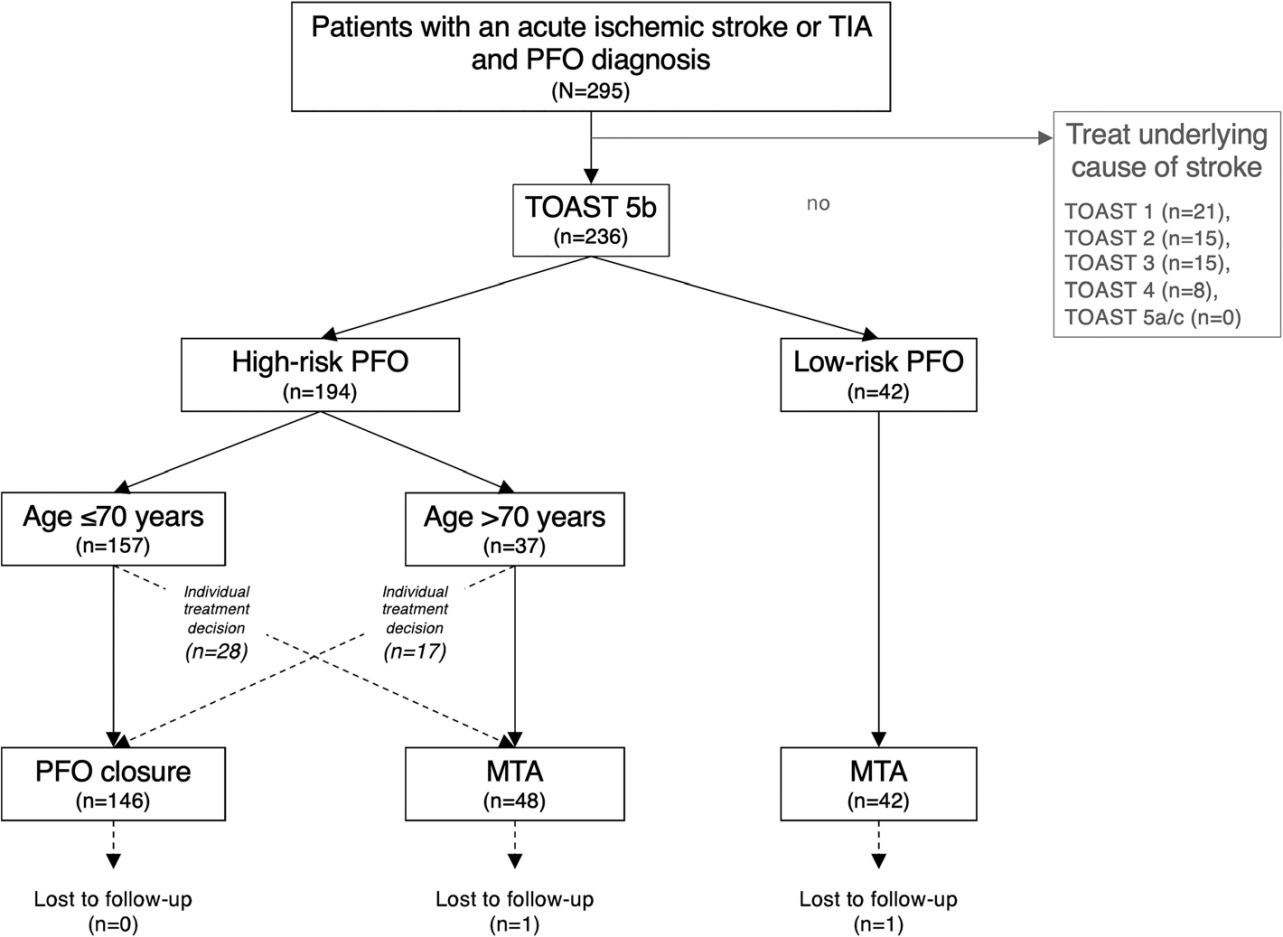
Selection algorithm for interventional and conservative PFO-management. Selection algorithm for interventional and conservative PFO-management according to standard operating procedure at our institution including the study flow with respective numbers. High-risk patent foramen ovale (PFO) is defined as PFO with either associated atrial septal aneurysm (ASA), spontaneous, or high-grade right-to-left shunt during Valsalva maneuver. Low-risk PFO is defined as PFO without ASA and with only small or moderate shunt size during Valsalva maneuver; MTA = medical therapy alone, TIA = transient ischemic attack, TOAST = Trial of Org 10172 in Acute Stroke Treatment

Recent RCT and epidemiological studies [17, 19, 26, 29, 30], as crystallized in recent international consensus recommendations [22, 24], have yielded progress with regard to causal attribution of PFO to IS/TIA, and the decision pro/con PFO-closure. However, no recommendations can be made for patients > 60 years of whom only very few were enrolled in one RCT [17, 30]. We established a standard operating procedure (SOP) at our institution, including a selection algorithm (Fig. 1) with an age-cutoff of 70 years for interventional/conservative PFO-management, and follow-up outcome assessment for at least 1 year. Our objective was to compare PFO-closure to secondary prevention with MTA in cryptogenic IS/TIA patients below and above 60 years.

**Fig. 2:**
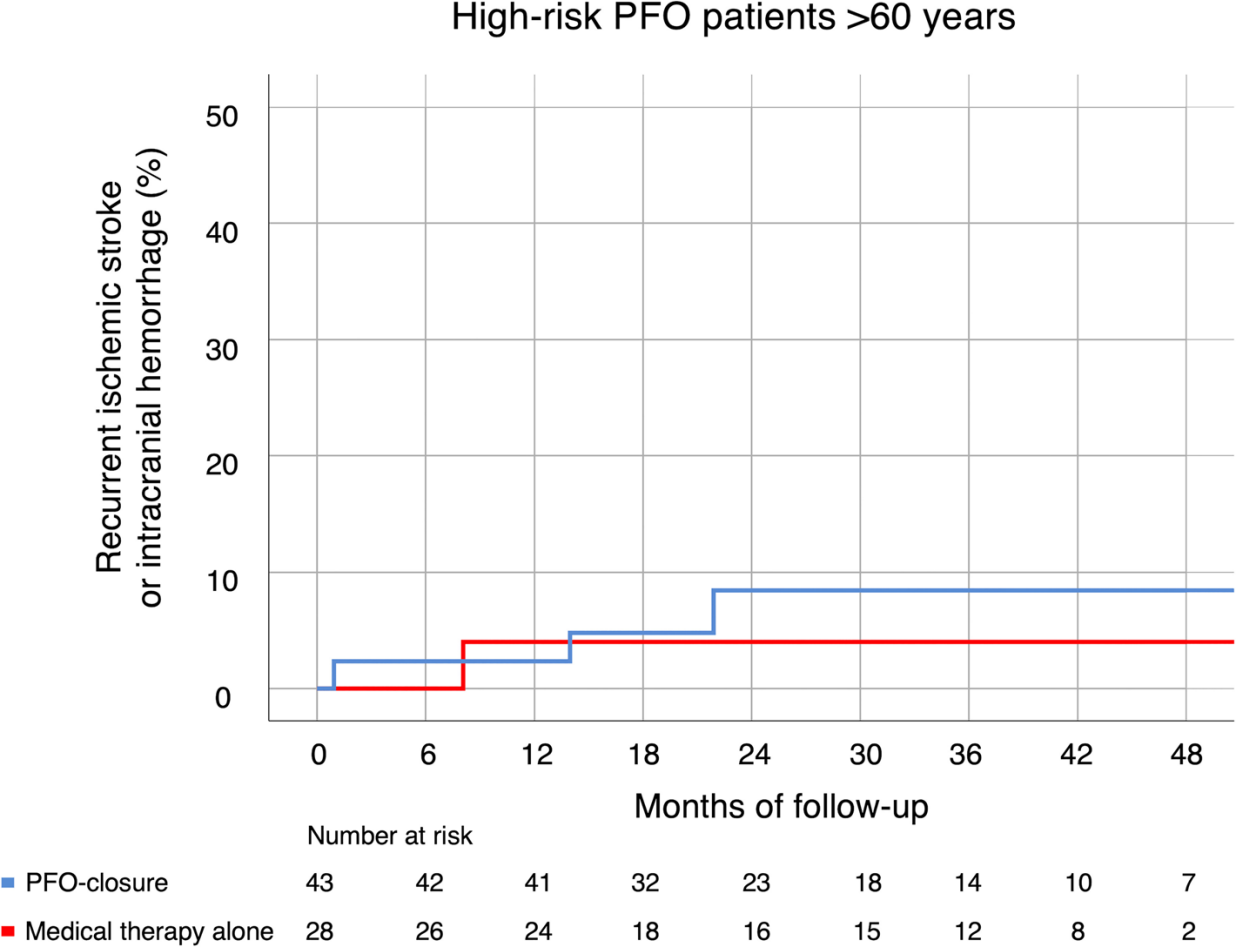
Cumulative recurrent stroke rates in high-risk PFO patients > 60 years. Cumulative event rates of recurrent ischemic stroke or intracranial hemorrhage in high-risk patent foramen ovale (PFO) patients above 60 years, who underwent ‘PFO-closure’ or were treated with ‘medical therapy alone’

## Methods

### Study design, protocol approval, and patients

Between March 2012 and September 2016 consecutive patients with PFO and IS/TIA of undetermined etiology (Trial of Org 10172 in Acute Stroke Treatment (TOAST) 5b [1]), were prospectively included in this single-center prospective case series at the department of neurology of Tuebingen university hospital, Germany, (http://www.clinicaltrials.gov. Unique identifier: NCT04352790). The study complies with the Declaration of Helsinki and was approved by the ethics committee at the Medical Faculty of Eberhard-Karls-University and University Hospital Tuebingen (protocol no. 522/2012BO2). Written informed consent was obtained from all patients or their legally authorized representatives prior to inclusion.

Please see Supplemental Methods for *Routine stroke work-up* and *Baseline assessment of clinical, echocardiographic and imaging parameters.*

### Selection algorithm, treatment, follow-up and endpoints

According to our SOP (Fig. 1), PFO-closure was recommended in patients ≤70 years with high-risk PFO, i.e., PFO with either associated ASA, spontaneous, or large right-to-left shunt during Valsalva maneuver. However, clinicians could depart from the recommended strategy for individual considerations.

Experienced interventional cardiologists performed PFO-closure with an Amplatzer™ PFO-occluder (25 or 35 mm; Abbott cardiovascular, Santa Clara, California, USA) after a median of 54 (IQR 41–104) days after index IS/TIA. Patients were bridged with direct oral anticoagulants (DOAC) or heparins if DOAC were contraindicated until PFO-closure. At PFO-closure, antithrombotic regimen was switched to dual antiplatelet therapy with aspirin and clopidogrel, or, in case of deep venous thrombosis or pulmonary embolism, clopidogrel was added to anticoagulation. During hospital stay for PFO-closure, patients were continuously monitored by telemetry to screen for periprocedural atrial fibrillation (AF); prolonged cardiac rhythm monitoring was not part of the study and left to the discretion of the treating physicians. Six weeks and 6 months after PFO-closure, patients presented at our outpatient clinic for follow-up contrast- transesophageal echocardiography to assess residual shunt and/or occluder-associated thrombi, infection, dislocation, or device erosion. If residual shunts and thrombi were excluded, patients were de-escalated to single antiplatelet therapy after three or 6 months, respectively.

Depending on shunt size, risk of paradoxical embolism (RoPE) score [13] on the one hand, and comorbidities, bleeding risk, and patient’s preference on the other hand, MTA consisted of either single antiplatelet therapy or oral anticoagulation, preferably with (off-label) DOAC.

In patients who underwent PFO-closure, long-term complications such as occluder-associated thrombi, infection, dislocation or device erosion were assessed and recorded during routine follow-up visits at the cardiology outpatient clinic. Additionally, a telephone follow-up was foreseen after ≥12 months for assessment of the primary endpoint, i.e., recurrent IS or intracranial hemorrhage, and secondary endpoints, i.e., degree of disability (modified Rankin Scale score; mRS), all-cause death, recurrent TIA, systemic embolisms, myocardial infarction, new-onset AF, major or clinically relevant non-major bleedings.

### Blinding

The telephone interviewer assessing all but device-related clinical endpoints at ≥12 months was blinded for all baseline characteristics including treatment, i.e., PFO-closure or MTA. To ensure blinding, at the very beginning of each conversation, patients or – in case patient was deceased or unable to communicate – relatives were instructed not to disclose treatment modality to the interviewer.

### Statistics

Continuous variables are expressed as mean and standard deviation or median with IQR. Categorical data are presented as proportions. Baseline variables were compared by standardized mean differences with > 0.2 indicating imbalance. Categorical primary and secondary outcome variables were analyzed by Fisher’s exact test. Relative risks (RR) and 95% confidence intervals (CI) were calculated for exploratory analyses. If covariates were missing, we imputed the mean values for continuous variables and the absence of the condition for dichotomous variables. Annual rates of IS recurrence were calculated by dividing total recurrence rate by mean follow-up time; in case median values were reported in the original publication, means were derived from those using the method described in [11]. Based on available RCT we planned subgroup analyses of patients ≤60 and > 60 years [6, 9, 17, 19, 20, 26, 29]. Comparison of the primary outcome was considered statistically significant for a two-sided *p*-value < 0.05; Bonferroni correction was applied to adjust for multiple testing of secondary outcomes. Statistical analyses were performed using IBM SPSS Statistics for Mac, v26 (IBM Corp., Armonk, NY, USA), and R v4.0.2 (R Foundation, Vienna, Austria). Reporting in accordance with STROBE guidelines for observational studies [31].

## Results

We prospectively screened 295 acute IS/TIA patients who were initially considered cryptogenic, consequently underwent transesophageal echocardiography and got diagnosed with PFO (Fig. 1). Of these, 236 (80%) were finally classified as TOAST 5b, i.e., IS/TIA of undetermined etiology, and were therefore included in the study.

### Patients with high-risk PFO undergoing PFO-closure

High-risk PFO were detected in 194 of the 236 included patients (82.2%). Of these, 129 patients ≤70 years and 20 patients > 70 years were treated in keeping with our SOP (Fig. 1). However, 28 patients ≤70 years and 17 patients > 70 years were crossovers, i.e., treated with the alternative strategy (please see Supplemental Results and Supplemental Tables 1 to 4); no patient with low-risk PFO was treated with ‘PFO-closure’.

Amplatzer™ PFO-occluders were used in all 146 ‘PFO-closure’ patients; 25 mm size in 132 cases. Implantation was unsuccessful in one patient (0.7%) due to extreme long-channel PFO. One patient suffered from periprocedural AF, which spontaneously ended within 24 h. No periinterventional clinically relevant or major bleeding occurred. Save for the patient where PFO-closure failed, all study subjects received follow-up transesophageal echocardiography 6 weeks after the intervention. Residual shunt was detected in 14 cases (9.6%; 12 small and two moderate). Device-associated thrombosis, infection, dislocation or device erosion were not observed. No long-term occluder-associated complications were noted.

### Patients receiving medical therapy alone

Overall, 90 patients received MTA. Of these, 42 had low-risk PFO of which 34 (81%) were treated with single antiplatelet therapy and eight (19%) with anticoagulants (seven DOAC, one phenprocoumon). Of the remaining 48 high-risk PFO patients who received MTA, 19 (40%) were treated with single antiplatelet therapy and 29 (60%) with anticoagulants (23 DOAC, six phenprocoumon).

### Comparison of different subgroups

#### PFO-closure patients ≤60 years vs. > 60 years

In the group of patients with high-risk PFO, we compared baseline characteristics of 103 ‘PFO-closure’ patients ≤60 years to those of 43 ‘PFO-closure’ patients > 60 years (Table 1). Unsurprisingly, (age-dependent) RoPE scores were higher in the younger group (median 7 vs. 4). The picture was mixed with regard to vascular risk factors: hypertension and previous IS/TIA were more common in older patients, but history of smoking, and coronary artery disease or myocardial infarction were less frequent. Interestingly, prevalence of ASA was higher in older patients.

**Table 1:** Patient baseline characteristics

										Standardized mean differences for comparison of			
	All (*N* = 236)	PFO-closure (*n* = 146)	MTA (*n* = 90)	PFO-closure ≤60 years + high-risk PFO (*n* = 103)	PFO-closure > 60 years + high-risk PFO (*n* = 43)	MTA ≤60 years + high-risk PFO (*n* = 20)	MTA > 60 years + high-risk PFO (*n* = 28)	MTA ≤60 years + low-risk PFO (*n* = 18)	MTA > 60 years + low-risk PFO (*n* = 24)	PFO-closure > 60 vs. ≤60 years	PFO-closure vs. MTA in high-risk PFO ≤60 years	PFO-closure vs. MTA in high-risk PFO > 60 years	low-risk vs. high-risk PFO
Age, years^a,b^	58 (47–66), 18–88	53 (45–62), 18–82	64 (52–75), 33–88	49 (40–54), 18–60	66 (63–73), 61–82	53 (41–58), 33–60	75 (69–79), 61–88	51 (47–55), 33–66	70 (65–76), 61–86	2.78	0.30	0.94	0.48
Sex, female^c^	91 (38.6)	59 (40.4)	32 (36)	45 (43.7)	14 (33)	9 (45)	9 (32)	7 (39)	7 (29)	0.23	0.03	0.01	0.13
Pre-stroke mRS 0^c^	221 (93.6)	138 (94.5)	83 (92)	100 (97.1)	38 (88)	20 (100)	25 (89)	16 (89)	22 (92)	0.34	0.25	0.03	0.15
*Medical history*													
Hypertension^c^	117 (49.6)	73 (50.0)	44 (49)	41 (39.8)	32 (74)	4 (20)	19 (68)	7 (39)	14 (58)	0.75	0.44	0.15	0.01
History of smoking^c^	56 (23.7)	37 (25.3)	19 (21)	33 (32.0)	4 (9)	5 (25)	5 (18)	7 (39)	2 (8)	0.59	0.16	0.25	0.07
Hyperlipidemia^c^	62 (26.3)	37 (25.3)	25 (28)	24 (23.3)	13 (30)	3 (15)	6 (21)	5 (28)	11 (46)	0.16	0.21	0.20	0.32
Obesity^c^	26 (11.0)	17 (11.6)	9 (10)	12 (11.7)	5 (12)	1 (5)	2 (7)	3 (17)	3 (13)	< 0.01	0.24	0.15	0.12
Diabetes^c^	26 (11.0)	9 (6.2)	17 (19)	5 (4.9)	4 (9)	2 (10)	6 (21)	3 (17)	6 (25)	0.17	0.20	0.34	0.36
CAD and/or prior MI^c^	15 (6.4)	5 (3.4)	10 (11)	5 (4.9)	0 (0)	0 (0)	2 (7)	2 (11)	6 (25)	0.32	0.32	0.39	0.50
Prior IS/TIA^c^	30 (12.7)	18 (12.3)	12 (13)	8 (7.8)	10 (23)	2 (10)	4 (14)	3 (17)	3 (13)	0.44	0.08	0.23	0.06
*Brain imaging*													
Acute ischemic lesions in multiple circulations^c^	15 (6.4)	7 (4.8)	8 (9)	5 (4.9)	2 (5)	1 (5)	4 (14)	2 (11)	1 (4)	0.01	0.01	0.33	0.04
*Echocardiography*													
Small shunt^c^	28 (11.9)	10 (6.8)	18 (20)	8 (7.8)	2 (5)	0 (0)	1 (4)	6 (33)	11 (46)	0.14	0.93	0.18	2.42
Moderate shunt^c^	65 (27.5)	32 (21.9)	33 (37)	23 (22.3)	9 (21)	0 (0)	8 (29)	12 (67)	13 (54)				
Large shunt^c^	143 (60.6)	104 (71.2)	39 (43)	72 (69.9)	32 (74)	20 (100)	19 (68)	0 (0)	0 (0)				
ASA^c^	99 (41.9)	75 (51.4)	24 (27)	47 (45.6)	28 (65)	3 (15)	21 (75)	0 (0)	0 (0)	0.40	0.71	0.22	1.44
LVEF, %^d^	59.2 ± 3.6	59.3 ± 3.7	59.1 ± 3.3	59.5 ± 3.1	58.7 ± 4.9	60.0 ± 0.0	57.7 ± 5.0	60.0 ± 0.0	59.4 ± 3.1	0.19	0.24	0.21	0.17
*Qualifying event*													
IS^c^	187 (79.2)	119 (81.5)	68 (76)	87 (84.5)	32 (74)	15 (75)	23 (82)	13 (72)	17 (71)	0.25	0.24	0.19	0.22
TIA^c^	49 (20.8)	27 (18.5)	22 (24)	16 (15.5)	11 (26)	5 (25)	5 (18)	5 (28)	7 (29)				
Admission NIHSS^a^	1 (0–4)	1 (0–4)	1 (0–4)	1 (0–4)	2 (0–4)	1 (0–2)	1 (0–3)	3 (0–6)	1 (0–5)	0.07	0.36	0.02	0.23
D-dimers > 0.5 μg/mL^c^	47 (19.9)	26 (17.8)	21 (23)	18 (17.5)	8 (19)	2 (10)	9 (32)	2 (11)	8 (33)	0.03	0.22	0.32	0.12
Deep vein thrombosis or pulmonary embolism^c^	12 (5.1)	7 (4.8)	5 (6)	4 (3.9)	3 (7)	2 (10)	1 (4)	1 (6)	1 (4)	0.14	0.24	0.15	0.02
RoPE score^a^	5 (4–7)	6 (4–7)	5 (3–6)	7 (5–8)	4 (3–5)	7 (6–7)	3 (3–4)	6 (5–7)	4 (3–5)	2.01	0.05	0.30	0.52

*ASA* atrial septal aneurysm, *CAD* coronary artery disease, *IS* ischemic stroke, *LVEF* left ventricular ejection fraction, *MI* myocardial infarction, *mRS* modified Rankin Scale score, *MTA* medical therapy alone, *n/a* not applicable, *NIHSS* National Institutes of Health Stroke Scale score, *PFO* patent foramen ovale, *RoPE* Risk of Paradoxical Embolism, *TIA* transient ischemic attack

^a^median (interquartile range), ^b^range, ^c^number (%), ^d^mean ± standard deviation

#### High-risk PFO patients ≤60 years: PFO-closure vs. ‘medical therapy alone’

In high-risk PFO patients ≤60 years who underwent PFO-closure, annual IS recurrence rate was 1.0% (Table 2, column 4), compared to annual rates of 1.5% in CLOSURE I [9], 0.1% in PC [20], 0.7% in RESPECT [6], 0% in CLOSE [19], 0.9% in Gore REDUCE [29], 1.2% in RESPECT EXT [26], and 0% in DEFENSE-PFO [17].

**Table 2:** Outcomes of patients with ‘patent foramen ovale (PFO)-closure’ and ‘medical therapy alone (MTA)’

										p for comparison of	Relative risk (95% confidence interval) for comparison of		
	All (*N* = 236)	PFO-closure (*n* = 146)	MTA (*n* = 90)	PFO-closure ≤60 years + high-risk PFO (*n* = 103)	PFO-closure > 60 years + high-risk PFO (*n* = 43)	MTA ≤60 years + high-risk PFO (*n* = 20)	MTA > 60 years + high-risk PFO (*n* = 28)	MTA ≤60 years + low-risk PFO (*n* = 18)	MTA > 60 years + low-risk PFO (*n* = 24)	PFO-closure > 60 vs. ≤60 years	PFO-closure vs. MTA in high-risk PFO ≤60 years	PFO-closure vs. MTA in high-risk PFO > 60 years	low-risk vs. high-risk PFO
Ischemic stroke^a^	7 (3.0)	6 (4.1)	1 (1)	3 (2.9)	3 (7)	0 (0)	1 (4)	0 (0)	0 (0)	0.36	1.41 (0.08–26.36)^§^	1.95 (0.21–17.85)	0.30 (0.02–5.19)^§^
Intracranial hemorrhage^a^	0 (0.0)									n/a			
Transient ischemic attack^a^	6 (2.5)	6 (4.1)	0 (0)	3 (2.9)	3 (7)	0 (0)	0 (0)	0 (0)	0 (0)	0.36^‡^	1.41 (0.08–26.36)^§^	4.61 (0.25–86.05)^§^	0.35 (0.02–6.08)^§^
Systemic embolism^a^	0 (0.0)									n/a			
Myocardial infarction^a^	3 (1.3)	2 (1.4)	1 (1)	1 (1.0)	1 (2)	0 (0)	1 (4)	0 (0)	0 (0)	0.50^‡^	0.61 (0.03–14.37)^§^	0.65 (0.04–9.99)	0.65 (0.03–12.31)^§^
Death from any cause^a^	10 (4.2)	2 (1.4)	8 (9)	1 (1.0)	1 (2)	0 (0)	4 (14)	1 (6)	3 (13)	0.50^‡^	0.61 (0.03–14.37)^§^	0.16 (0.02–1.38)	3.08 (0.91–10.43)
New-onset atrial fibrillation^a^	1 (0.4)	1 (0.7)	0 (0)	0 (0.0)	1 (2)	0 (0)	0 (0)	0 (0)	0 (0)	0.29^‡^	n/a	1.98 (0.08–46.89)^§^	1.51 (0.06–36.48)^§^
Major Bleedings^a^	0 (0.0)									n/a			
Major or clinically relevant non-major bleedings^a^	0 (0.0)									n/a			
PFO-unrelated outcome events^a^	14 (5.9)	5 (3.4)	9 (10)	2 (1.9)	3 (7)	0 (0)	5 (18)	1 (6)	3 (13)	0.15^‡^	1.01 (0.05–20.28)^§^	0.49 (0.12–2.02)	2.05 (0.66–6.35)
MRS at follow-up 0 or 1^a^	198 (83.9)	129 (88.4)	69 (77)	96 (93.2)	33 (77)	20 (100)	19 (68)	0 (0–1)	0 (0–2)	0.01^‡^	3.03 (0.18–51.02)^§^	0.72 (0.34–1.55)	2.13 (1.17–3.87)
										Standardized mean differences			
Follow-up time (days)^b^	1016 ± 479	1013 ± 471	1021 ± 494	1061 ± 471	899 ± 455	1114 ± 433	878 ± 474	993 ± 457	1131 ± 570	0.35	0.12	0.05	0.14

*MRS* modified Rankin Scale score, *n/a* not applicable

^a^number (%), ^b^mean ± standard deviation, ‡secondary outcomes: after Bonferroni adjustment, *p* < 0.005 is considered significant, §0.5 was added to each group if zero events in one group

The 20 high-risk PFO patients ≤60 years who received MTA (all crossovers) had similar RoPE scores compared to the 103 high-risk PFO patients ≤60 years who underwent PFO-closure (Table 1). Besides higher prevalence of deep venous thrombosis or pulmonary embolism, former were ‘healthier’ with regard to pre-stroke mRS, vascular risk factors, D-dimers, and admission National Institutes of Health Stroke Scale (NIHSS) score, and no outcome event was detected in this group.

#### High-risk PFO patients > 60 years: PFO-closure vs. ‘medical therapy alone’

When comparing high-risk PFO patients > 60 years who underwent PFO-closure (*n* = 43) with those high-risk PFO patients > 60 years receiving MTA (*n* = 28), the higher age of the latter (median 75 vs. 66 years), which is an obvious result of our selection algorithm (Fig. 1), combined with higher rates of diabetes and history of smoking, well explains lower RoPE scores in this group (median 3 vs. 4) (Table 2). Overall, vascular risk factors – except previous IS/TIA – but also elevated D-dimers and ASA were more common in the MTA group. IS recurrence rate being 7% (95%CI 0.0–16.4) in high-risk PFO patients > 60 years who underwent PFO-closure compared to 4% (95%CI 0.0–12.0) in those receiving MTA (RR 1.95, 95%CI 0.21–17.85) (Table 2 and Fig. 2). Interestingly, all recurrent IS were again classified as TOAST 5b (please see Supplemental Table 5). Rate of outcome events unrelated to PFO (i.e., all events except ischemic stroke, TIA, systemic embolism, and related death) was 7% (95%CI 0.0–15.6) in high-risk PFO patients > 60 years who underwent PFO-closure and 18% (95%CI 4.6–32.3) in high-risk PFO patients > 60 years who received MTA (RR 0.49, 95%CI 0.12–2.02).

#### High-risk vs. low-risk PFO patients

Compared to the 194 high-risk PFO patients, the 42 low-risk PFO patients were older, had more vascular risk factors, and consequently lower RoPE scores. Low-risk PFO patients had higher admission NIHSS scores and more often TIA (rather than IS) as qualifying event. All 42 low-risk PFO patients were treated with MTA and none of them suffered recurrent IS. Of the eight patients who were treated with oral anticoagulants, five had deep venous thrombosis, pulmonary embolism or suspected hypercoagulable state and three received oral anticoagulation due to high RoPE score. Rate of outcome events unrelated to PFO was 10% (95%CI 2.2–19.6) in low-risk and 5.2% (95%CI 2.5–8.7) in high-risk PFO patients (RR 2.05, 95%CI 0.66–6.35). Low-risk PFO patients less likely reached an mRS of 0 or 1 at follow-up.

Overall, no intracranial hemorrhage, systemic embolism, or major or clinically relevant non-major bleeding occurred. Ten patients died during follow-up, three from cardiovascular events other than stroke, and seven from non-cardiovascular disease. Further outcome measures and follow-up times are summarized in Table 2. Two MTA patients (one with high-risk, and one with low-risk PFO) were lost to follow-up with no information available after discharge (Fig. 1).

## Discussion

In this prospective case series including consecutive patients with PFO who presented with acute cryptogenic IS/TIA (TOAST 5b) at our stroke unit, we evaluated a straightforward SOP to guide treatment decisions regarding interventional or conservative PFO-management.

To date, six RCT investigating transcatheter PFO-closure for secondary stroke prevention in patients with cryptogenic IS/TIA have been published, five of them with an upper age limit of 60 years [6, 9, 17, 19, 20, 26, 29]. Data from beyond this age is thus scarce. Taking a closer look at patient selection, baseline characteristics and (sub) group effects in the different trials, the benefit of PFO-closure is clearly demonstrated for patients ≤60 years and can be attributed to the inclusion of patients with high-risk PFO [17, 19, 29]; longer duration of follow-up increases the positive effect of PFO-closure seen in the trials [26].

Our prospectively collected real-world data of patients with PFO and cryptogenic IS/TIA confirms these findings, which is unsurprising as patient selection for ‘PFO-closure’ resembles inclusion criteria of the more recent RCT [17, 19, 29]. However, we indicated PFO-closure also in high-risk PFO patients > 60 years of age. This allowed us to estimate recurrent IS rates in patients who were not assessed in RCT so far – except the very few patients enrolled in DEFENSE-PFO [17]. Physicians should be wary of just extrapolating trial results to an older population: Multimorbidity in older patients (compare Table 1) may indicate that competing other-than-PFO etiologies may underly the qualifying or recurrent IS/TIA, which might be better addressed by an adequate MTA. On the other hand, elderly patients are more likely to suffer deep vein thrombosis, pulmonary emboli and right cardiac pressure overload which renders a PFO more susceptible for paradoxical trans-cardiac emboli.

PFO-closure would only be expected to prevent recurrent PFO-related events. Notably, in none of our patients suffering recurrent IS another-than-PFO cause could be identified (please see Supplemental Table 5). Too little precision in our estimates, however, does not allow any firm conclusion on optimal treatment of high-risk PFO patients > 60 years (compare Table 2 and Fig. 2). As PFO-associated embolism is supposed to be caused either by a paradoxical embolus originating from the venous system or an embolus formed in or at the atrial septum or ASA, antiplatelet therapy may not be the right choice for prevention of recurrent embolism. A recent meta-analysis of NAVIGATE ESUS, PICSS and the CLOSE trial indicates that anticoagulation compared to antiplatelet therapy might reduce the risk of stroke recurrence among cryptogenic stroke patients with PFO by about half [12]. In our study, patients with high-risk PFO were predominantly treated with oral anticoagulation, preferably with DOAC, depending on their age, either as bridging therapy until PFO-closure (≤70 years) or as life-long MTA (> 70 years). No patient with low-risk PFO underwent PFO-closure and only the minority (19%) received oral anticoagulation due to either a high RoPE score or venous thrombosis/suspected hypercoagulable state. Although the numbers and events of patients who received oral anticoagulation or antiplatelet therapy in our study was limited, the observed 0% IS/TIA recurrence indicates that the use of the latter might be sufficiently effective for secondary stroke prevention in the majority of patients with low-risk PFO. However, no bleeding events were noted during follow-up in either treatment group. Higher mortality in MTA patients (compared to our patients who were treated with PFO-closure) was unrelated to stroke and reflects their older age and higher overall morbidity.

In our study, the rate of successful device implantation was 99%, and of residual shunt 9.6%. This confirms the similarly high rates of procedural success observed in recent RCT and indicates feasibility of the intervention also in patients > 60 years when using modern devices [17, 19, 29]. Importantly, two of our patients with residual shunt suffered IS/TIA during follow-up.

We detected (transient) periprocedural AF in only one patient (0.6%), and no patient reported AF detection during follow-up. Comparable rates of periprocedural AF were observed in PC and DEFENSE-PFO, i.e., two out of 204 and one out of 60 patients [17, 20]. Other RCT did not distinguish periprocedural AF from new-onset AF during follow-up [6, 9, 19, 26, 29]. In prior studies [2, 4, 28], up to 20% of new-onset AF after PFO-closure led to the suggestion that the procedure itself induces AF. Postinterventional AF has also been associated to the type of occluder, with the STARFlex™ (NMT Medical, Boston, Massachusetts, USA) showing higher AF rates than the Amplatzer™ PFO-occluder [9, 20]. The sole use of Amplatzer™ PFO-occluders in our patients may have contributed to the low prevalence of new-onset AF. Besides, reporting of AF during follow-up was based on a telephone interview and not on repetitive Holter recordings or loop recorders [4]. On the one hand, AF after PFO-closure is usually transient and the clinical relevance remains uncertain [9, 29]. On the other hand, paroxysmal AF was detected in a relevant number of cryptogenic stroke patients and might constitute the actual cause underlying the index or recurrent strokes [23, 25]. Due to its therapeutic implications, prolonged cardiac rhythm monitoring might thus contribute to the exclusion of occult AF also in patients with cryptogenic stroke and PFO.

### Limitations

The decision for interventional PFO-closure or MTA was not randomized. Nonetheless, real-world data on outcome of patients with cryptogenic IS/TIA undergoing PFO-closure or MTA are of considerable clinical value as they may verify the findings of RCT. Second, due to the single-center setting and the relatively short follow-up interval, the number of patients and of total events is rather low. A-priori sample size calculation was not conducted. Third, the telephone interviewer did not use a standardized, validated recurrent IS/TIA questionnaire to determine whether new events had occurred. However, medical records of all patients reporting events were obtained from treating hospitals. Fourth, comparison of the rates of recurrent IS and intracranial hemorrhage in our patients ≤60 years with RCT data is limited due to the different duration of follow-up. In RCT, time of follow-up ranges between 2 years in CLOSURE I and nearly 6 years in RESPECT EXT. [9, 26] However, we calculated annual rates of recurrent IS for all trials to facilitate comparison. Fifth, we only used Amplatzer™ PFO-occluders, whereas CLOSURE I, CLOSE, and Gore REDUCE used different types of PFO-occluders [9, 19, 29]. Sixth, the number of new-onset AF after PFO-closure may be underestimated in our study as systematic prolonged cardiac monitoring > 72 h was not performed. However, prolonged rhythm monitoring was equally not required in neither of the six RCT nor in NAVIGATE or RESPECT ESUS [6, 8, 9, 12, 17, 19, 20, 29]. We did not assess aortic plaque load in our study, a relevant condition which might have significantly contributed to stroke recurrence risk, especially in elderly patients. Optimal antithrombotic regimen for treatment of aortic arch atherosclerosis, however, is still under debate [3]. Importantly, all patients of our study received an antithrombotic. Finally, many high-risk PFO patients were not treated according SOP but with the alternative strategy (Fig. 1). Individual treatment decision of such a relevant fraction inevitably leads to selection bias and independently of baseline characteristics (please see Supplemental Results).

## Conclusion

The 1.0% annual stroke recurrence rate in our high-risk PFO patients ≤60 years who underwent ‘PFO-closure’ replicates the results of the six available RCT [6, 9, 17, 19, 20, 26, 29] in a real-world setting. High-risk PFO patients > 60 years who underwent PFO-closure had similar IS recurrence rates (7%) than those treated with MTA (4%; RR 1.95, 95%CI 0.21–17.85). Finally, the low stroke recurrence rate in MTA-treated low-risk PFO patients of any age indicates the appropriateness of this therapeutic choice in this population.

## Supplementary Information

**Additional file 1.**

## Data Availability

The datasets used and/or analyzed during the current study are available from the corresponding author on reasonable request.

## Abbreviations

AF: Atrial fibrillation
ASA: Atrial septal aneurysm
CAD: Coronary artery disease
CI: Confidence interval
DOAC: Direct oral anticoagulant(s)
IS: Ischemic stroke
LVEF: Left ventricular ejection fraction
MI: Myocardial infarction
MTA: Medical therapy alone
mRS: Modified Rankin Scale score
n/a: Not applicable
NIHSS: National Institutes of Health Stroke Scale
PFO: Patent foramen ovale
RCT: Randomized controlled trial(s)
RoPE: Risk of Paradoxical Embolism
TIA: Transient ischemic attack
TOAST: Trial of Org 10172 in Acute Stroke Treatment
