# Supplementary material for: Closure or medical therapy of patent foramen ovale in cryptogenic stroke: prospective case series
Source: Neurol Res Pract. 2021 Apr 1;3:16. doi: 10.1186/s42466-021-00114-3 (PMC8015114; doi:10.1186/s42466-021-00114-3)
Supplement: Supplementary file 1 — Additional file 1. [file 42466_2021_114_MOESM1_ESM.docx]

**Supplementary Material**

**Supplemental Methods**

*Routine stroke work-up*

All patients underwent a thorough neurological assessment of the underlying cause of ischemic stroke (IS)/transient ischemic attack (TIA).

IS/TIA etiologies were categorized according to the “Trial of Org 10172 in Acute Stroke Treatment“ (TOAST) criteria (Adams et al., 1993); in case of TIA with the limitation of missing information on acute ischemic lesion pattern.(Amort et al., 2012)

The diagnostic work-up included brain imaging with computed tomography and additional magnetic resonance imaging in patients with no visible infarct on computed tomography. Cases in whom diffusion weighted magnetic resonance imaging revealed no ischemic lesion, and symptoms lasted less than 24 hours, were categorized as TIA. Vessel imaging included either computed tomography angiography or MR angiography, and ultrasound of extra- and intracranial brain-supplying arteries. Cardiac evaluation included transthoracic echocardiography, and, due to undetermined stroke etiology, prolonged cardiac rhythm monitoring for at least 72 hours, blood sampling for assessment of hypercoagulable states, and deep vein ultrasound in case D-dimer levels were above normal. Additionally, and only in case of clinical suspicion, blood screening tests for vasculitis and cerebral spinal fluid analysis were performed. Due to its invasive nature, transesophageal echocardiography was restricted to cryptogenic IS/TIA patients who were considered eligible for PFO closure or start of anticoagulation during hospital stay, i.e., patients with less severe stroke symptoms and smaller infarcts.

*Baseline assessment of clinical, echocardiographic and imaging parameters*

At baseline, age, sex, the National Institutes of Health Stroke Scale (NIHSS) score, modified Rankin Scale score (mRS), D-dimer above normal, presence of deep vein thrombosis, and vascular risk factors were assessed including obesity, diabetes, hypertension, hyperlipidemia, history of smoking, (paroxysmal) atrial fibrillation, coronary artery disease or history of myocardial infarction, and prior IS/TIA.

The following echocardiographic parameters were recorded: presence of atrial septal aneurysm (ASA; defined as excursion of the septal tissue of > 10 mm from the plane of the atrial septum into the right or left atrium or a combined total excursion right and left of 15 mm),(Silvestry et al., 2015) semiquantitative shunt size (small if 3–9, moderate if 10–30, and large if >30 contrast bubbles appeared in the left atrium),(Mas et al., 2001) detection of intracardiac thrombi and left ventricular ejection fraction estimated by biplane Simpson’s method. Presence of acute infarcts in multiple circulations was assessed by brain imaging. The Risk of Paradoxical Embolism (RoPE) score (range 0 to 10, with higher scores equaling a higher probability that PFO is related to stroke) was calculated as previously described.(Kent et al., 2013)

**Supplemental Results**

*Comparison of crossover vs. non-crossover high-risk PFO patients ≤70 years*

Compared to the 129 high-risk PFO patients ≤70 years who were treated with PFO-closure according SOP (non-crossovers), the 28 crossover patients who received MTA instead were older but had lower pre-stroke mRS, less vascular risk factors, and, thus, similar RoPE scores (see Supplemental Table I). Larger shunts were more and ASA less common in the crossover patients; more often, they had TIA rather than IS as qualifying event, and lower NIHSS on admission.

17 (61%) crossover patients opted against PFO-closure, whereas treating physicians did not recommend PFO-closure in eleven (39%). Five of the latter required anticoagulation due to deep vein thrombosis or suspected hypercoagulable state, and another-than-PFO, yet, undetermined embolic source (e.g., large but non-stenotic large‐artery atherosclerosis) was thought to underly index IS/TIA in the remaining six patients. Half of the crossovers underwent antiplatelet therapy and the other half underwent anticoagulation.

Notably, no outcome events were observed in the 28 crossover patients (see Supplemental Table II).

*Comparison of crossover vs. non-crossover high-risk PFO patients >70 years*

Compared to the 20 high-risk PFO patients >70 years who received MTA according SOP (non-crossovers), the 17 crossover patients who underwent PFO-closure instead were younger (see Supplemental Table III). Except obesity and deep vein thrombosis, vascular risk factors were either similar or less frequent in the group of crossovers. Crossovers had larger shunts and higher RoPE scores, but less often ASA; they also had more often TIA as qualifying event, but their admission NIHSS was higher than that of non-crossovers.

Seven (41%) crossover patients insisted on PFO-closure. In ten (59%) cases, however, the treating physicians recommended PFO-closure over anticoagulation; five crossovers were considered biologically younger, three had high bleeding risk, and two concomitant disease requiring long-term antiplatelet therapy. For outcome events see Supplemental Table IV.

**Supplemental Tables**

**Supplemental Table 1: Baseline characteristics of crossover and non-crossover patients with high-risk patent foramen ovale (PFO) and age less or equal 70 years**

|  | Crossover patients:  high-risk PFO  ≤70 years + MTA  (n=28) | Non-crossover patients:  high-risk PFO  ≤70 years + PFO-closure  (n=129) | Standardized mean differences for comparison of  crossover vs. non-crossover patients |
| --- | --- | --- | --- |
| Age, years^*,†^ | 55 (44–62), 33–70 | 51 (43–59), 18–70 | 0.34 |
| Sex, female^‡^ | 12 (43) | 51 (39.5) | 0.07 |
| Pre-stroke mRS 0^‡^ | 28 (100) | 123 (95.3) | 0.31 |
| *Medical history* |  |  |  |
| Hypertension^‡^ | 8 (29) | 60 (46.5) | 0.38 |
| History of smoking^‡^ | 7 (25) | 35 (27.1) | 0.05 |
| Hyperlipidemia^‡^ | 3 (11) | 34 (26.4) | 0.41 |
| Obesity^‡^ | 2 (7) | 15 (11.6) | 0.15 |
| Diabetes^‡^ | 3 (11) | 8 (6.2) | 0.16 |
| CAD and/or prior MI^‡^ | 0 (0) | 5 (3.9) | 0.28 |
| Prior IS/TIA^‡^ | 2 (7) | 15 (11.6) | 0.15 |
| *Brain imaging* |  |  |  |
| Acute ischemic lesions in multiple circulations^‡^ | 1 (4) | 5 (3.9) | 0.02 |
| *Echocardiography* |  |  |  |
| Small shunt^‡^ | 0 (0) | 10 (7.8) | 0.62 |
| Moderate shunt^‡^ | 2 (7) | 27 (20.9) |  |
| Large shunt^‡^ | 26 (93) | 92 (71.3) |  |
| ASA^‡^ | 9 (32) | 64 (49.6) | 0.36 |
| LVEF^§^ | 59.8±0.9 | 59.3±3.4 | 0.21 |
| *Qualifying event* |  |  |  |
| IS^‡^ | 20 (71) | 105 (81.4) | 0.24 |
| TIA^‡^ | 8 (29) | 24 (18.6) |  |
| Admission NIHSS^*^ | 1 (0–2) | 1 (0–3) | 0.41 |
| D-dimers >0.5 µg/mL^‡^ | 5 (18) | 22 (17.1) | 0.02 |
| Deep vein thrombosis or pulmonary embolism^‡^ | 2 (7) | 5 (3.9) | 0.14 |
| RoPE score^*^ | 6 (5–7) | 6 (5–7) | 0.09 |

ASA=atrial septal aneurysm, CAD=coronary artery disease, IS=ischemic stroke, LVEF=left ventricular ejection fraction, MI=myocardial infarction, mRS=modified Rankin Scale score, MTA=medical therapy alone, n/a=not applicable, NIHSS=National Institutes of Health Stroke Scale score, RoPE=Risk of Paradoxical Embolism, TIA=transient ischemic attack. *median (interquartile range), †range, ‡number (%), §mean ± standard deviation

**Supplemental Table 2: Outcomes of crossover and non-crossover patients with high-risk patent foramen ovale (PFO) and age less or equal 70 years**

|  | Crossover patients:  high-risk PFO  ≤70 years + MTA  (n=28) | Non-crossover patients:  high-risk PFO  ≤70 years + PFO-closure  (n=129) | Relative risk (95% confidence interval) for comparison of crossover vs. non-crossover patients |
| --- | --- | --- | --- |
| Ischemic stroke^*^ | 0 (0) | 3 (2.3) | 0.64 (0.03–12.06)^‡^ |
| Intracranial hemorrhage^*^ | 0 (0) | 0 (0) | n/a |
| Transient ischemic attack^*^ | 0 (0) | 5 (3.9) | 0.41 (0.02–7.17)^‡^ |
| Systemic embolism^*^ | 0 (0) | 0 (0) | n/a |
| Myocardial infarction^*^ | 0 (0) | 1 (0.8) | 1.49 (0.06–35.76)^‡^ |
| Death from any cause^*^ | 0 (0) | 1 (0.8) | 1.49 (0.06–35.76)^‡^ |
| New-onset atrial fibrillation^*^ | 0 (0) | 0 (0) | n/a |
| Major Bleedings^*^ | 0 (0) | 0 (0) | n/a |
| Major or clinically relevant non-major bleedings^*^ | 0 (0) | 0 (0) | n/a |
| PFO-unrelated outcome events^*^ | 0 (0) | 2 (1.6) | 0.90 (0.04–18.18)^‡^ |
| mRS at follow-up 0 or 1^*^ | 28 (100) | 117 (90.7) | 0.18 (0.01–2.94)^‡^ |
|  |  |  |  |
|  |  |  | Standardized mean differences |
| Follow-up time (days)^†^ | 1104±429 | 1034±463 | 0.16 |

MRS=modified Rankin Score, MTA=medical therapy alone, n/a=not applicable. *number (%), †mean ± standard deviation, ‡0.5 was added to each group if zero events in one group.

**Supplemental Table 3: Baseline characteristics of crossover and non-crossover patients with high-risk patent foramen ovale (PFO) and age above 70 years**

|  | Crossover patients:  high-risk PFO  >70 years + PFO-closure (n=17) | Non-crossover patients:  high-risk PFO  >70 years + MTA  (n=20) | Standardized mean differences for comparison of  crossover vs.  non-crossover patients |
| --- | --- | --- | --- |
| Age, years^*,†^ | 74 (71–76), 71–82 | 78 (74–81), 72–88 | 0.97 |
| Sex, female^‡^ | 8 (47) | 6 (30) | 0.36 |
| Pre-stroke mRS 0^‡^ | 15 (88.2) | 17 (85) | 0.10 |
| *Medical history* |  |  |  |
| Hypertension^‡^ | 13 (76) | 15 (75) | 0.03 |
| History of smoking^‡^ | 2 (12) | 3 (15) | 0.10 |
| Hyperlipidemia^‡^ | 3 (18) | 6 (30) | 0.29 |
| Obesity^‡^ | 2 (12) | 1 (5) | 0.25 |
| Diabetes^‡^ | 1 (6) | 5 (25) | 0.55 |
| CAD and/or prior MI^‡^ | 0 (0) | 2 (10) | 0.47 |
| Prior IS/TIA^‡^ | 3 (18) | 4 (20) | 0.06 |
| *Brain imaging* |  |  |  |
| Acute ischemic lesions in multiple circulations^‡^ | 2 (12) | 4 (20) | 0.23 |
| *Echocardiography* |  |  |  |
| Small shunt^‡^ | 0 (0) | 1 (5) | 0.33 |
| Moderate shunt^‡^ | 5 (29) | 6 (30) |  |
| Large shunt^‡^ | 12 (71) | 13 (65) |  |
| ASA^‡^ | 11 (65) | 15 (75) | 0.23 |
| LVEF, %^§^ | 58.8±5.7 | 57.0±5.7 | 0.32 |
| *Qualifying event* |  |  |  |
| IS^‡^ | 14 (32) | 18 (90) | 0.22 |
| TIA^‡^ | 3 (18) | 2 (10) |  |
| Admission NIHSS^*^ | 2 (0–4) | 1 (0–5) | 0.03 |
| D-dimers >0.5 µg/mL^‡^ | 4 (24) | 6 (30) | 0.15 |
| Deep vein thrombosis or pulmonary embolism^‡^ | 2 (12) | 1 (5) | 0.25 |
| RoPE score^*^ | 3 (3–4), 3–5 | 3 (3–4), 1–5 | 0.42 |

ASA=atrial septal aneurysm, CAD=coronary artery disease, IS=ischemic stroke, LVEF=left ventricular ejection fraction, MI=myocardial infarction, mRS=modified Rankin Scale score, MTA=medical therapy alone, n/a=not applicable, RoPE=Risk of Paradoxical Embolism, TIA=transient ischemic attack. *median (interquartile range), †range, ‡number (%), §mean ± standard deviation

**Supplemental Table 4: Outcomes of crossover and non-crossover patients with high-risk patent foramen ovale (PFO) and age above 70 years**

|  | Crossover patients:  high-risk PFO  >70 years + PFO-closure (n=17) | Non-crossover patients:  high-risk PFO  >70 years + MTA  (n=20) | Relative risk (95% confidence interval) for comparison of crossover vs. non-crossover patients |
| --- | --- | --- | --- |
| Ischemic stroke^*^ | 3 (18) | 1 (5) | 3.53 (0.40–30.88) |
| Intracranial hemorrhage^*^ | 0 (0) | 0 (0) | n/a |
| Transient ischemic attack^*^ | 1 (6) | 0 (0) | 3.50 (0.15–80.71)^‡^ |
| Systemic embolism^*^ | 0 (0) | 0 (0) | n/a |
| Myocardial infarction^*^ | 1 (6) | 1 (5) | 1.176 (0.08–17.42) |
| Death from any cause^*^ | 1 (6) | 4 (20) | 0.29 (0.04–2.39) |
| New-onset atrial fibrillation^*^ | 1 (6) | 0 (0) | 3.50 (0.15–80.71)^‡^ |
| Major Bleedings^*^ | 0 (0) | 0 (0) | n/a |
| Major or clinically relevant non-major bleedings^*^ | 0 (0) | 0 (0) | n/a |
| PFO-unrelated outcome events^*^ | 3 (18) | 5 (25) | 0.88 (0.23–3.40) |
| MRS at follow-up 0 or 1^*^ | 12 (71) | 11 (65) | 0.65 (0.27–1.58) |
|  |  |  |  |
|  |  |  | Standardized mean differences |
| Follow-up time (days)^†^ | 851±513 | 797±471 | 0.11 |

MRS=modified Rankin Score, MTA=medical therapy alone, n/a=not applicable. *number (%), †mean ± standard deviation, ‡0.5 was added to each group if zero events in one group.

**Supplemental Table 5: Patients with recurrent strokes during follow-up**

| Patient | Index event | Recurrent stroke | Description of recurrent stroke |
| --- | --- | --- | --- |
| #1 (female, age 80, high-risk PFO, RoPE score 3, MTA) | Ischemic stroke on January 11^th^ 2015 (TOAST 5b) | Ischemic stroke on September 9^th^ 2015 (TOAST 5b) | Cryptogenic,  noncompliance with regard to antithrombotic therapy |
| #2 (age 76, high-risk PFO, RoPE score 5, PFO-closure) | Ischemic stroke on December 18^th^ 2015 (TOAST 5b) | Ischemic stroke on January 15^th^ 2016 (TOAST 5b) | Cryptogenic |
| #3 (age 75, high-risk PFO, RoPE score 4, PFO-closure) | Ischemic stroke on January 1^st^ 2015 (TOAST 5b) | Ischemic stroke on February 29^th^ 2016 (TOAST 5b) | Cryptogenic, loop recorder implanted, but no AF detected during 3 years of monitoring |
| #4 (age 73, high-risk PFO, RoPE score 3, PFO-closure) | Ischemic stroke on November 13^th^ 2014 (TOAST 5b) | Transient ischemic attack on August 15^th^ 2015 (TOAST 5b), and ischemic stroke on August 31^st^ 2016 (TOAST 5b) | Cryptogenic, loop recorder implanted, but no AF detected during 3 years of monitoring |
| #5 (age 38, high-risk PFO, RoPE score 7, PFO-closure) | Ischemic stroke on August 9^th^ 2013 (TOAST 5b) | Ischemic stroke on August 15^th^ 2016 (TOAST 5b) | Cryptogenic, implantation of a second PFO occluder due to residual shunt detected at the date of recurrent stroke |
| #6 (age 42, high-risk PFO, RoPE score 8, PFO-closure) | Ischemic stroke on March 31^st^ 2014 (TOAST 5b) | Ischemic stroke on May 27^th^ 2014 (TOAST 5b) | Cryptogenic, switched to direct oral anticoagulant due to suspected thrombophilia, despite normal extended coagulation workup |
| #7 (age 56, high-risk PFO, RoPE score 7, PFO-closure) | Ischemic stroke on November 1^st^ 2012 (TOAST 5b) | Ischemic strokes on March 28^th^ 2013 and on July 24^th^ 2014 (both TOAST 5b) | Cryptogenic at time of recurrent event. However, switched to vitamin K antagonist in 2018 after recurrent deep vein thromboses and diagnosis of heterozygous factor V Leiden mutation |

AF=atrial fibrillation, MTA=medical therapy alone, PFO=patent foramen ovale, RoPE=Risk of paradoxical embolism, TOAST=Trial of Org 10172 in Acute Stroke Treatment

**Supplemental References**

Adams, H. P., Jr., Bendixen, B. H., Kappelle, L. J., Biller, J., Love, B. B., Gordon, D. L., & Marsh, E. E., 3rd. (1993, Jan). Classification of subtype of acute ischemic stroke. Definitions for use in a multicenter clinical trial. TOAST. Trial of Org 10172 in Acute Stroke Treatment. *Stroke, 24*(1), 35-41. <http://www.ncbi.nlm.nih.gov/pubmed/7678184>

Amort, M., Fluri, F., Weisskopf, F., Gensicke, H., Bonati, L. H., Lyrer, P. A., & Engelter, S. T. (2012). Etiological classifications of transient ischemic attacks: subtype classification by TOAST, CCS and ASCO--a pilot study. *Cerebrovasc Dis, 33*(6), 508-516. <https://doi.org/10.1159/000337236>

Kent, D. M., Ruthazer, R., Weimar, C., Mas, J. L., Serena, J., Homma, S., Di Angelantonio, E., Di Tullio, M. R., Lutz, J. S., Elkind, M. S., Griffith, J., Jaigobin, C., Mattle, H. P., Michel, P., Mono, M. L., Nedeltchev, K., Papetti, F., & Thaler, D. E. (2013, Aug 13). An index to identify stroke-related vs incidental patent foramen ovale in cryptogenic stroke. *Neurology, 81*(7), 619-625. <https://doi.org/10.1212/WNL.0b013e3182a08d59>

Mas, J. L., Arquizan, C., Lamy, C., Zuber, M., Cabanes, L., Derumeaux, G., & Coste, J. (2001, Dec 13). Recurrent cerebrovascular events associated with patent foramen ovale, atrial septal aneurysm, or both. *N Engl J Med, 345*(24), 1740-1746. <https://doi.org/10.1056/NEJMoa011503>

Silvestry, F. E., Cohen, M. S., Armsby, L. B., Burkule, N. J., Fleishman, C. E., Hijazi, Z. M., Lang, R. M., Rome, J. J., Wang, Y., American Society of, E., Society for Cardiac, A., & Interventions. (2015, Aug). Guidelines for the Echocardiographic Assessment of Atrial Septal Defect and Patent Foramen Ovale: From the American Society of Echocardiography and Society for Cardiac Angiography and Interventions. *J Am Soc Echocardiogr, 28*(8), 910-958. <https://doi.org/10.1016/j.echo.2015.05.015>
